# Limited mitogenomic degradation in response to a parasitic lifestyle in Orobanchaceae

**DOI:** 10.1038/srep36285

**Published:** 2016-11-03

**Authors:** Weishu Fan, Andan Zhu, Melisa Kozaczek, Neethu Shah, Natalia Pabón-Mora, Favio González, Jeffrey P. Mower

**Affiliations:** 1 Center for Plant Science Innovation, University of Nebraska, Lincoln, NE 68588, USA; 2 Department of Agronomy and Horticulture, University of Nebraska, Lincoln, NE 68583, USA; 3 Department of Computer Sciences and Engineering, University of Nebraska, Lincoln, NE 68588, USA; 4 Instituto de Biología, Universidad de Antioquia, Apartado 1226, Medellín, Colombia; 5 Facultad de Ciencias, Instituto de Ciencias Naturales, Universidad Nacional de Colombia, Apartado 7495, Sede Bogotá, Colombia

## Abstract

In parasitic plants, the reduction in plastid genome (plastome) size and content is driven predominantly by the loss of photosynthetic genes. The first completed mitochondrial genomes (mitogenomes) from parasitic mistletoes also exhibit significant degradation, but the generality of this observation for other parasitic plants is unclear. We sequenced the complete mitogenome and plastome of the hemiparasite *Castilleja paramensis* (Orobanchaceae) and compared them with additional holoparasitic, hemiparasitic and nonparasitic species from Orobanchaceae. Comparative mitogenomic analysis revealed minimal gene loss among the seven Orobanchaceae species, indicating the retention of typical mitochondrial function among Orobanchaceae species. Phylogenetic analysis demonstrated that the mobile *cox1* intron was acquired vertically from a nonparasitic ancestor, arguing against a role for Orobanchaceae parasites in the horizontal acquisition or distribution of this intron. The *C. paramensis* plastome has retained nearly all genes except for the recent pseudogenization of four subunits of the NAD(P)H dehydrogenase complex, indicating a very early stage of plastome degradation. These results lend support to the notion that loss of *ndh* gene function is the first step of plastome degradation in the transition to a parasitic lifestyle.

One of the defining characteristics of plants is the presence of a plastid, which enables the fixation of carbon to produce organic molecules via photosynthesis. Parasitic plants represent a dramatic departure from the typical autotrophic lifestyle of plants because they obtain organic carbon sources heterotrophically, using specialized organs called haustoria to make direct connections with the vascular tissue in the roots or shoots of a host plant. Parasitic plants, which comprise approximately 1% of all angiosperms^1^, can be subdivided based on the extent of their reliance on heterotrophy: hemiparasites retain the ability to photosynthesize and obtain only some of their nutrients from their hosts, while holoparasites have lost photosynthetic ability and must obtain all of their nutrition from hosts.

The transition from an autotrophic to a heterotrophic lifestyle has had a dramatic impact on the plastid genome (plastome) of parasitic plants. Studies of parasitic plant plastomes have established a wide range of genomic degradation, defined primarily by the presence or absence of photosynthetic activity and degree of dependence of the host. For example, in Orobanchaceae, the facultative hemiparasite *Triphysaria versicolor* has not lost any plastid genes, while the obligate hemiparasites *Schwalbea americana* and *Striga hermonthica* (also in Orobanchaceae) exhibit minimal pseudogenization/loss of only a few *ndh* genes, which encode subunits of the plastid NAD(P)H dehydrogenase complex^2^,^3^. Plastomes of hemiparasitic mistletoes (Viscaceae) are slightly more degraded, exhibiting both a reduction in size (down to 126–147 kb) and the loss of all 11 *ndh* genes plus a small number (1–6) of non-photosynthetic genes^4^. Within *Cuscuta* (Convolvulaceae), the four sequenced plastomes range from 85 to 125 kb in size and have experienced more extensive gene loss, yet they still retain all (or all but one) photosynthetic genes^5^,^6^, which is consistent with at least low levels of photosynthetic activity^7^. Other *Cuscuta* species are clearly non-photosynthetic and their plastomes have lost numerous photosynthetic and non-photosynthetic genes^7^,^8^. Plastomes in the holoparasitic species of Orobanchaceae are also heavily degraded^2^,^3^,^9^,^10^,^11^, most extensively in *Conopholis americana* whose plastome is only 46 kb in size with just 21 intact protein-coding genes. Similar levels of degradation were found in the plastomes of other holoparasites in Cynomoriaceae and Hydnoraceae^12^,^13^. Even greater genomic reduction was reported in *Pilostyles* (Apodanthaceae), whose plastomes are reduced to just 11–15 kb and may contain only five or six functional genes^14^. In some holoparasites, such as *Rafflesia lagascae* (Rafflesiaceae), the entire plastome may have been lost^15^.

Much less is known about the effects of a parasitic lifestyle on the mitochondrial genomes (mitogenomes) of plants. In fact, only a single genus of parasitic plants has a completely sequenced mitogenome, from the hemiparasitic mistletoes *Viscum scurruloideum* and *Viscum album*, along with draft genomes from two additional *Viscum* species^16^,^17^. Compared with other land plants, *V. scurruloideum* has the smallest mitogenome (66 kb) and all four *Viscum* sequences have lost functional copies of all nine *nad* genes encoding subunits of the mitochondrial NADH dehydrogenase complex I, the first reported loss of this complex from any multicellular eukaryote^16^. In contrast, the draft mitogenome from the holoparasite *R. lagascae* has a typical size (estimated at > 300 kb) for an angiosperm and contains a nearly complete set of protein-coding genes, including at least seven of nine *nad* genes^15^. The draft mitogenome of *Cynomorium coccineum* is even larger (>1 Mb) and also contains a nearly complete set of mitochondrial genes^13^. Thus, the effect of a plant parasitic lifestyle on the mitogenome is still unclear, requiring the analysis of mitogenomes from additional parasitic lineages.

Despite the limited mitogenomic information for parasitic plants, it is well established that their mitochondrial DNA undergoes frequent horizontal transfer, which is likely facilitated by the direct physical connection between parasitic and host plants^18^,^19^,^20^. Perhaps the best studied example of plant horizontal transfer involves the mobile group I intron of the cytochrome oxidase subunit 1 (*cox1*) gene. This intron was originally acquired from fungi and has been subsequently transferred many times during angiosperm evolution^21^,^22^,^23^,^24^. Intriguingly, this *cox1* intron is highly overrepresented in the parasitic plants that have been examined to date, suggesting that parasitic plants may serve as mediators of horizontal intron transfer among angiosperms^25^. Although this hypothesis was not supported in an analysis with limited sampling of parasitic plants^25^, denser sampling of parasites and closely related nonparasitic taxa is needed before the hypothesis should be rejected.

The Orobanchaceae is an ideal family for studies on parasitic plant evolution because it contains the full range of trophic specialization, including a nonparasitic lineage (*Lindenbergia*), numerous hemiparasitic lineages with varying degrees of photosynthetic activity and host dependence (e.g., *Bartsia*, *Castilleja*, *Schwalbea*, *Striga*), and at least three transitions to holoparasitism (e.g., *Lathraea*, *Orobanche*, *Hyobanche*) resulting in a complete loss of photosynthesis^26^,^27^,^28^. Complete plastome sequences are available from more than a dozen species in Orobanchaceae, but only a few are from hemiparasites, while data from the mitogenome in this family is lacking. To improve our understanding of organellar genomic evolution in hemiparasitic plants, we sequenced the complete mitochondrial and plastid genomes from the facultative hemiparasite *Castilleja paramensis*. Furthermore, to assess mitogenomic diversity within the Orobanchaceae, we generated draft mitogenome sequences from six additional species representing the range of trophic diversity: the autotroph *Lindenbergia philippensis*, the hemiparasites *Bartsia pedicularioides* and *S. americana*, and the holoparasites *Orobanche crenata*, *Orobanche gracilis*, and *Phelipanche ramosa*. These sequences were compared to assess the degree of genomic degradation in response to a parasitic lifestyle.

## Results

### The mitochondrial genome of the hemiparasite *Castilleja paramensis*

**Figure 1:**
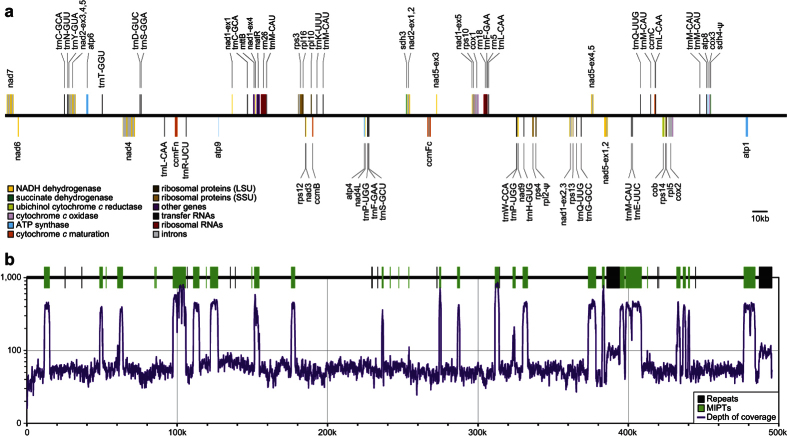
The *Castilleja paramensis* mitogenome. (**a**) Gene and intron map. Top genes are transcribed in the forward direction; bottom genes are transcribed in the reverse direction. Colors correspond to the functional categories listed in the key. (**b**) Correlation of repeats and MIPTs with depth of sequencing coverage. The location of all repeats (black) and MIPTs (green) >100 bp in length are shown. Genome maps were drawn with OgDraw (http://ogdraw.mpimp-golm.mpg.de/).

The complete mitogenome of *C. paramensis* maps as a single circular chromosome that is 495,499 bp in length (Fig. 1A). The genome includes a total of 67 genes (34 protein-coding, 3 rRNA, and 30 tRNA) and 23 introns (17 *cis-*spliced and 6 *trans-*spliced). In addition to these functional elements, repeats and MIPTs (mitochondrial DNA of plastid origin) comprise a substantial component of this genome (Fig. 1B). There is one large repeat of 8.7 kb, 15 intermediate repeats from 100 to 447 bp, and 32 small repeats between 50 and 100 bp. Together, these repeats cover 2.7% (13,525 bp) of the genome. A total of 43 MIPTs are also present. Ranging in size from 116 bp to 7.7 kb, these MIPTs cover 16.6% (82,133 bp) of the mitogenome, which is the highest MIPT percentage observed in any plant yet sequenced. The MIPTs contain 55 full-length or nearly full-length plastid genes, about half of which are pseudogenes based on the presence of frameshifting indels and/or premature stop codons. With the exception of the MIPTs and repeats, the depth of sequencing coverage is consistently at ~50x throughout most portions of the mitogenome. The greatly increased coverage depth at most MIPTs is likely due to mismapping of reads in the data set that were derived from the plastome. The two-fold increase in coverage depth of the 8.7 kb repeat relative to the rest of the mitogenome is an indication that this region is in fact present in two copies in the genome, consistent with its status as a repeat.

### Limited gene and intron loss from the parasitic Orobanchaceae mitogenomes

**Figure 2:**
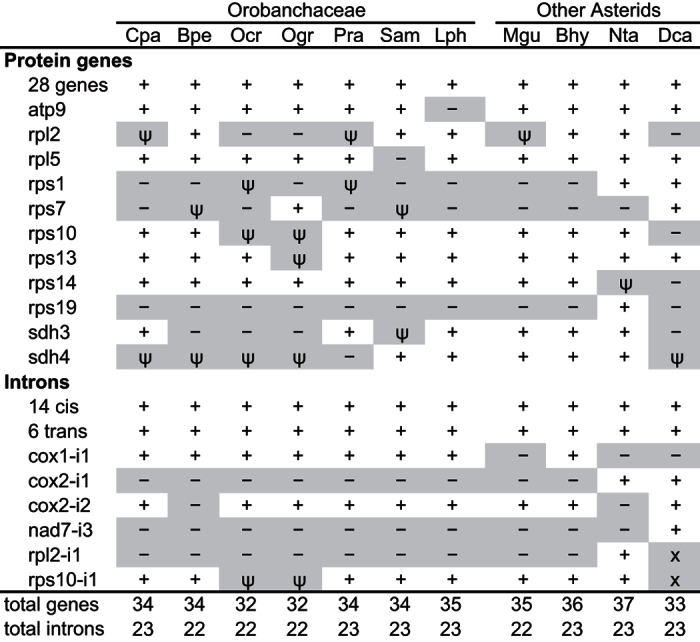
Mitochondrial gene and intron content in Orobanchaceae and selected asterids. Genes and introns present in each genome are marked with a plus symbol (“+”). Lost genes and introns (“−”), pseudogenized genes and introns (“ψ”), and missing introns due to loss of the host gene (“x”) are shaded gray. The 28 genes include *atp1*, *atp4*, *atp6*, *atp8*, *ccmB*, *ccmC*, *ccmFc*, *ccmFn*, *cob*, *cox1*, *cox2*, *cox3*, *matR*, *mttB*, *nad1*, *nad2*, *nad3*, *nad4*, *nad4L*, *nad5*, *nad6*, *nad7*, *nad9*, *rpl10*, *rpl16*, *rps3*, *rps4*, and *rps12*. The 14 *cis*-arranged introns include *ccmFc*-i1, *nad1*-i2, *nad2*-i1, *nad2*-i3, *nad2*-i4, *nad4*-i1, *nad4*-i2, *nad4*-i3, *nad5*-i1, *nad5*-i4, *nad7*-i1, *nad7*-i2, *nad7*-i4, and *rps3*-i1. The six *trans*-arranged introns include *nad1*-i1*, nad1*-i3*, nad1*-i4, *nad2*-i2*, nad5*-i2, and *nad5*-i3. Cpa = *Castilleja paramensis*; Bpe = *Bartsia pedicularioides*; Ocr = *Orobanche crenata*; Ogr = *Orobanche gracilis*; Pra = *Phelipanche ramosa*; Sam = *Schwalbea americana*; Lph = *Lindenbergia philippensis*; Mgu = *Mimulus guttatus*; Bhy = *Boea hygrometrica*; Nta = *Nicotiana tabacum*; Dca = *Daucus carota*.

In contrast to the extensive mitochondrial gene and intron loss observed in mistletoes, there is only minor variation in gene and intron content in Orobanchaceae (Fig. 2), based on comparative mitogenomic analysis of a nonparasite (*L. philippensis*), three hemiparasites (*B. pedicularioides*, *C. paramensis*, *S. americana*), and three holoparasites (*O. crenata, O. gracilis, P. ramosa*). The mitogenomes of all seven Orobanchaceae species share 29 protein-coding genes. This conserved set encompasses 23 of the 24 core genes that are nearly universally present in angiosperm mitogenomes^29^, including nine subunits for the NADH dehydrogenase complex (*nad1*, *2*, *3*, *4*, *4L*, *5*, *6*, *7*, *9*), the apocytochrome *b* gene for the cytochrome *bc*_1_ complex (*cob*), three subunits for the cytochrome *c* oxidase complex (*cox1*, *2*, *3*), four of the five subunits for the ATP synthase complex (*atp1*, *4*, *6*, *8*), the four cytochrome *c* maturation factors (*ccmB*, *C*, *Fc*, *Fn*), an intron maturase (*matR*), and a protein translocase (*mttB/tatC*). For the remaining ATP synthase subunit (*atp9*), the gene is present in all species except *L. philippensis*. However, the lack of detection of this very short gene (only 225 bp) should be interpreted with caution because it could be an artefact of an incomplete draft assembly.

There is more variability in the presence of genes encoding subunits of the ribosomal protein and succinate dehydrogenase complexes among the Orobanchaceae species (Fig. 2). Six protein members of the large (*rpl10*, *16*) and small (*rps3*, *4*, *12*, *14*) ribosomal subunits are conserved in all seven Orobanchaceae mitogenomes, whereas the remaining seven ribosomal proteins and both succinate dehydrogenase genes have been lost or pseudogenized in at least one species. Also, several genes (*O. gracilis rps7*, *O. crenata* and *P. ramosa rps13*, *C. paramensis* and *L. philippensis sdh3*) have been tentatively scored as present and putatively functional in this study, although they are truncated by 20–30% and may be pseudogenes. Further analysis is required to assess whether they retain functionality. The other listed pseudogenes exhibit clearer loss-of-function mutations because they are heavily truncated (*O. crenata rps1*, *O. gracilis rps10* and *rps13*, *P. ramosa rpl2* and *rps1*, *S. americana rps7*) or they have one or more frameshifts (*O. crenata rps10* and *sdh4*, *O. gracilis sdh4*, *S. americana sdh3*) that cannot be attributed to pyrosequencing errors at mononucleotide repeats.

In terms of intron content, all examined Orobanchaceae species contain either 22 or 23 introns (Fig. 2). In all seven Orobanchaceae species, there are 15 introns removed by *cis* splicing and 6 by *trans* splicing. All seven species lack *cox2*-i1, *nad7*-i3, and *rpl2*-i1, as do other sequenced Lamiales species (e.g., *Boea*, *Mimulus*), suggesting that the introns were lost early in Lamiales evolution prior to the radiation of Orobanchaceae. Within the Orobanchaceae, the *cox2*-i2 intron was uniquely lost from *B. pedicularioides*, while in the *rps10* pseudogenes from *O. crenata* and *O. gracilis*, remnants of the *rps10* intron are still retained.

### The Orobanchaceae *cox1* intron was acquired vertically from a non-parasitic ancestor

Previous studies have identified the mobile *cox1* intron in a small fraction of angiosperms, between 4% and 25% of the hundreds of examined species in the two most extensive analyses^22^,^23^. In contrast to the general scarcity of this intron among angiosperms, it was previously observed that a large fraction of parasitic plants (15 out of 17 examined species, representing 12 distinct parasitic lineages) possess the intron, including *Epifagus virginiana*, the only Orobanchaceae parasite to be examined thus far^25^. In agreement with this observation, this intron is present in all six parasitic Orobanchaceae species examined in the current study, and also in the nonparasitic *L. philippensis* (Fig. 2).

The mobile nature of the *cox1* intron, coupled with the overrepresentation of this intron in parasitic plants and the fact that parasitic plants are known to facilitate the horizontal transfer of mitochondrial DNA among species^18^,^19^,^20^, raises two possibilities: 1) parasitic plants may frequently transfer this intron to other angiosperms, explaining the abundant horizontal transmission of the intron among angiosperms, and 2) parasitic plants may frequently acquire this intron from other angiosperms, explaining the overrepresentation of the intron in parasitic plants. Both hypotheses can be tested phylogenetically. If parasitic plants are frequent donors of the intron to other angiosperms, then we would expect to find the introns of recipient angiosperms nested within the parasitic plant clade of introns. If parasitic plants are frequently receiving the intron from other angiosperms, then we would expect to see the horizontally acquired introns of parasitic plants cluster with the donating angiosperm clades rather than in the expected organismal position for Orobanchaceae species within Lamiales.

**Figure 3:**
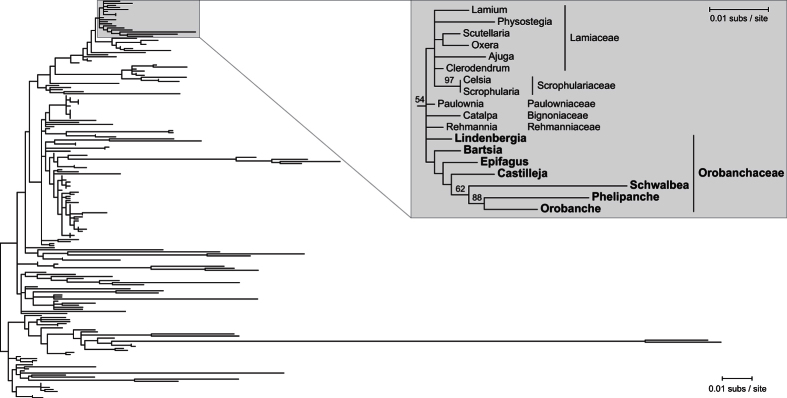
Phylogenetic analysis of the mobile *cox1* intron. The tree results from maximum likelihood evaluation of 194 intron sequences from diverse angiosperms. The expanded section of the tree depicts a clade of Lamiales sequences from the seven Orobanchaceae species (large, bold text) and closely related families. Family names are labeled to the right of the subtree. Bootstrap values ≥ 50% from 500 replicates are shown on the branch. The subtree is drawn to a 2-fold expanded scale relative to the full tree; scale bars for the subtree and full tree are shown at top right and bottom right, respectively.

Phylogenetic analysis of the *cox1* intron from Orobanchaceae sequences and a diverse collection of other angiosperms demonstrates that neither hypothesis is correct for the parasitic plants in this family (Fig. 3; Figure S1). Within the tree, there is a clade that comprises all Orobanchaceae parasites, which is nested within a larger clade of Lamiales that includes the nonparasitic *Lindenbergia*, also from Orobanchaceae, plus other species (*Catalpa*, *Paulownia, Rehmannia*) from families that are closely related to the Orobanchaceae. Support for most relationships within this larger Lamiales clade is generally weak (<50% bootstrap support for most branches). Nevertheless, the monophyletic clustering of Orobanchaceae species in the more-or-less expected position within Lamiales indicates that this *cox1* intron was most likely acquired vertically in parasitic Orobanchaceae from a nonparasitic ancestor. Furthermore, the absence of any unexpected species nested within the parasitic Orobanchaceae clade indicates that the parasites did not donate the *cox1* intron to any of the other angiosperm species sampled in the analysis.

### Minimal degeneration of the *Castilleja paramensis* plastid genome

**Figure 4:**
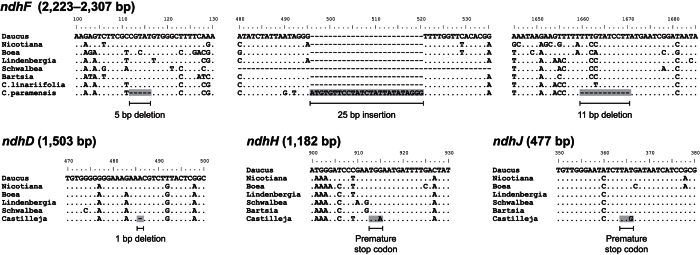
Evidence for pseudogenization of *Castilleja paramensis ndh* genes. Shown are sections of four *ndh* gene alignments with evidence of pseudogenization. The frameshifting indels and premature stop codons leading to loss of function are shaded in gray. The full length of functional versions of each gene is shown in parentheses next to each gene name. C. = *Castilleja*.

The *C. paramensis* plastome (Figure S2) is 152,926 bp in length, with a typical quadripartite structure that includes the large and small single-copy regions separated by two copies of an inverted repeat. Relative to the gene and intron content present in a typical asterid, the *C. paramensis* plastome contains nearly a full set of protein-coding genes, a full set of 4 rRNAs and 31 tRNAs, and a full set of 21 introns (Figure S2 and S3). The few exceptions involve the pseudogenization of *ndhD* and *ndhF* due to frameshifting indels and *ndhH* and *ndhJ* due to the presence of premature stop codons (Fig. 4). The pseudogenization of *ndhF* does not occur in all *Castilleja* species, as an intact gene was sequenced from *Castilleja linariifolia* in a previous study^30^. Like *C. paramensis*, the obligate hemiparasite *S. americana* has also lost functionality of several *ndh* genes (pseudogenization of *ndhA*, *ndhD*, *ndhF*, *ndhG*, *ndhJ* and loss of *ndhI*), whereas Orobanchaceae holoparasites including *Cistanche*, *Conopholis*, and *Orobanche* have lost ~70% of all of their genes, including nearly all of the photosynthesis-related genes and numerous tRNAs (Figure S3)^3^,^10^,^11^. Similar patterns of minor degeneration in hemiparasites and more extensive degeneration in holoparasites were observed in a recent broad analysis of Orobanchaceae species^2^.

## Discussion

### Gene loss from Orobanchaceae mitogenomes is unrelated to parasitism

In this study, we generated one complete mitogenome from the hemiparasite *C. paramensis* and draft mitogenomes from six additional Orobanchaceae species, including two more hemiparasites (*B. pedicularioides* and *S. americana*), three holoparasites (*O. crenata, O. gracilis* and *P. ramosa*), and a nonparasite (*L. philippensis*). Despite the wide range of trophic strategies among the examined Orobanchaceae species, their mitogenomes display no evidence of functional degeneration that can be attributed to the adoption of a parasitic lifestyle. The relatively few mitochondrial genes that were lost or pseudogenized are limited to ribosomal proteins and succinate dehydrogenase subunits (Fig. 2). The loss of these genes is not attributable to the adoption of a parasitic lifestyle because these same genes have also been lost from the mitogenomes of many non-parasitic land plants^16^,^29^,^31^,^32^. Importantly, their loss is unlikely to have a detrimental effect on mitochondrial activity, as each loss event is usually preceded by the establishment of a homolog in the nucleus that maintains a functional product^33^,^34^,^35^,^36^. Like in these other examples, we suggest that the functions of the missing Orobanchaceae mitochondrial genes have been supplanted by nuclear-encoded homologs, although sequencing of the nuclear genome will be required to test this prediction.

In addition to the Orobanchaceae data reported here, large-scale mitogenomic data from a parasitic plant is available from four hemiparasitic mistletoes^16^,^17^, four holoparasitic members of Rafflesiaceae^15^,^37^, and a holoparasite in Cynomoriaceae^13^. As in the Orobanchaceae parasites described here, the Cynomoriaceae and Rafflesiaceae holoparasites contain a nearly complete set of the expected mitochondrial genes, although a substantial fraction were reported to have been acquired horizontally^13^,^37^. By contrast, in the hemiparasitic *V. scurruloideum*, the mitogenome has been greatly reduced in size, and in all four mistletoes the coding content has undergone extreme reduction, including the pseudogenization or loss of all nine *nad* genes encoding subunits of mitochondrial complex I, a NADH dehydrogenase^16^,^17^. The coordinated loss of functional copies of all nine *nad* genes, which has not been reported for any other multicellular eukaryote, argues against a nuclear transfer scenario and instead suggests that the entire complex I was lost^16^, with nuclear-encoded alternative dehydrogenases^38^ compensating for the loss of complex I activity.

Thus, while the reduced mitochondrial sequences from mistletoes suggested the possibility of general mitochondrial upheaval in parasitic plants, this does not appear to be the case, at least in the members of Orobanchaceae examined here or in the members of Rafflesiaceae and Cynomoriaceae examined previously. Overall, based on the available data from parasitic plants, there does not appear to be any clear correlation between mitogenomic degradation and the degree of host dependence. This is perhaps not surprising as the mitochondrion is essential for respiration and the production of ATP, and these processes are still required by parasitic plants to generate amino acids and other essential organic molecules. The putative loss of complex I from *Viscum* may reflect the first step in mitogenomic degradation in a parasitic plant, which may be tolerated due to the partially overlapping abilities of the alternative dehydrogenases^16^. Regardless, the unusual mitogenomic features observed for *Viscum* are clearly not representative of all parasitic plants. Whether this complex or any other seemingly essential mitochondrial genes have been lost in other parasitic lineages awaits further investigation.

### No evidence that the *cox1* intron was acquired or distributed horizontally by Orobanchaceae parasites

Studies have indicated that the angiosperm *cox1* intron was acquired from a fungal donor and then horizontally transferred numerous times among species, evidenced primarily by the sporadic distribution of the intron among species and extensive phylogenetic incongruence in the intron tree^21^,^22^,^23^,^24^. Barkman *et al*. made the intriguing observation that nearly all examined parasitic plants possess this intron, but they found no evidence that the intron was acquired from their putative hosts^25^. Alternatively, parasitic plants, particularly those with nonspecific host preferences, may serve as key players in the horizontal spread of the intron.

Using the multiple Orobanchaceae *cox1* introns assembled in this study, we demonstrate in this study that the Orobanchaceae introns were acquired vertically from a nonparasitic ancestor (Fig. 3; Figure S1), consistent with the initial results of Barkman *et al*. using a single Orobanchaceae intron sequence^25^. Furthermore, there is no evidence that the Orobanchaceae parasites facilitated the spread of the intron to any of the other intron-containing species included in the phylogeny. Overall, there are no indications that the parasitic lifestyle has had any influence on the presence of the *cox1* intron in Orobanchaceae or its transfer from Orobanchaceae to any of the other species that were included in the analysis. Thus, it remains unclear why parasitic plants tend to have the *cox1* intron, or whether the proclivity of parasitic plants for horizontal transfer plays any role in the intron’s spread. Broad sampling from additional parasitic plant lineages may help to shed light on any potential connections between the parasitic lifestyle and the distribution *cox1* intron.

### Plastid genome degeneration in parasitic plants

Unlike the mitogenome of *C. paramensis*, which exhibits few signs of functional degradation, the *C. paramensis* plastome has frameshift mutations or premature stop codons in four subunits of the plastid NAD(P)H dehydrogenase complex (Fig. 4). These mutations in the well-conserved *ndh* genes are likely to lead to a reduction or loss of gene function. This pattern of *ndh-*specific degradation in the *C. paramensis* plastome is similar to observations in other Orobanchaceae hemiparasites and some species of *Cuscuta* (Figure S3)^2^,^3^,^5^,^6^. The draft plastome from the hemiparasite *Bartsia inaequalis* also lacks intact, full-length copies of several *ndh* genes (*ndhD*, *ndhE*, *ndhG*, and *ndhI*), although it cannot be ruled out that these genes were missed due to the incomplete nature of the genome^39^. Compared with other sequenced hemiparasites, the *C. paramensis* plastome appears to be in the very earliest stages of degradation, as indicated by the small number of genes so-far affected, the limited number of deleterious mutations that have accumulated in each affected gene and the lack of any genes that were deleted completely. Furthermore, an intact *ndhF* gene is present in another *Castilleja* species^30^, indicating that the pseudogenization of the *C. paramensis ndhF* gene occurred recently within the genus, at some point after *C. paramensis* diverged from other members of the genus. The *C. paramensis* plastome thus provides strong support for the idea that loss of the NAD(P)H dehydrogenase complex is the first step of plastome degradation in the evolution of heterotrophy in plants^3^,^40^. By contrast, the plastomes from nonphotosynthetic holoparasites are generally much more degraded than those of hemiparasites, affecting not only the full spectrum of photosynthetic genes but also many genes not directly related to photosynthesis (Figure S3)^2^,^3^,^8^,^9^. Taken together, the collective evidence from available parasitic plastomes suggests a connection between the degree of plastomic degeneration and heterotrophic dependence.

Although it is possible that these genes have been functionally transferred to the nuclear genome in *C. paramensis*, there has been no demonstration of functional *ndh* gene transfer for any seed plants that have lost the plastid *ndh* genes. Fragments of some *ndh* genes were identified in the nucleus of several Orobanchaceae species^11^, but there is no indication that these fragments produce functional proteins. Instead, mounting evidence in multiple lineages—including the pine family, gnetophytes, several orchids, and several species of *Erodium* (Geraniaceae)—has shown that these lost plastid genes were not relocated to the nucleus, and furthermore, that many of the nuclear-encoded subunits of this complex have also been lost^41^. These results strongly suggest that the entire NAD(P)H complex has been eliminated from these species.

## Materials and Methods

### Sample collection and organellar genome sequencing

A *C. paramensis* individual was collected from a páramo in the department of Boyacá, Colombia on March 21, 2014 (voucher *N. Pabón-Mora et al. 299*, HUA). A *B. pedicularioides* individual was collected from a páramo in Cajas National Park, Ecuador on December 17, 2010 (voucher *J. P. Mower et al. 2064*, QCA). Total genomic DNA was extracted from silica-dried leaves using the Plant DNeasy Kit (Qiagen). DNA samples were sequenced on the Illumina HiSeq2000 platform at BGI (Shenzhen, China), which generated 6 Gb (for *B. pedicularioides*) or 8 Gb (for *C. paramensis*) of 100-bp paired-end reads from an 800-bp library.

### Genome assembly and annotation

Draft organellar genomes of *C. paramensis* and *B. pedicularioides* were assembled from the Illumina sequence reads with Velvet version 1.2.03^42^ using multiple combinations of kmer (61, 71, 81, 91) and expected coverage (50, 100, 200, 500, 1000) values, as described previously^43^,^44^. Organellar contigs were identified in each assembly by using default blastn searches with known organellar gene sequences from related Lamiales species as queries. For each targeted genome, the best assembly that maximized total mitochondrial or plastid length in the fewest number of contigs was used for further scaffolding. Scaffolding was performed by mapping read pairs onto the contig sequences using blastn (e-value ≤ 1 × 10^−10^, hit length ≥ 90 bp, sequence identity ≥ 90%), and read pairs spanning two different contigs were used to infer contig joins and repeat regions. Using this strategy, circular-mapping plastid and mitochondrial genomes were assembled for *C. paramensis*, and a draft mitogenome was assembled for *B. pedicularioides*. The *C. paramensis* and *B. pedicularioides* mitogenome assemblies were annotated as described previously^32^,^44^,^45^. The *C. paramensis* plastid genome was annotated using DOGMA^46^ followed by manual adjustment as necessary.

To survey mitochondrial gene content in additional Orobanchaceae species, 454 pyrosequencing data from a previous study^47^ were downloaded from the NCBI sequence read archive (accession SRA047928) for one hemiparasite (*S. americana*), three holoparasites (*O. crenata*, *O. gracilis*, *P. ramosa*) and one nonparasite (*L. philippensis*). The downloaded 454 data were assembled with Velvet 1.2.03 as described above using various pairwise combinations of kmer (41, 51, 61, 71) and expected coverage (5, 10, 20, 50, 100) values. Lower kmer and expected coverage values were required for these data sets given the lower amount of data available (<1 Gb total genomic DNA for each species), resulting in assemblies with 5–10x depth of mitochondrial sequence coverage for each species. Scaffolding was not performed because the reads were unpaired. The presence of mitochondrial genes and introns was scored by using blastn searches with mitochondrial gene sequences from other Lamiales species as queries against the best 454 assemblies. Gene and intron sequences of interest were manually extracted from these 454 assemblies for further analysis.

Genes identified from each assembly were assessed for potential loss of function by searching for frameshifting indels and/or premature stop codons. Genes were scored as pseudogenes if the mutations disrupted >20% of their conserved domain structure, as defined by a search of the NCBI Conserved Domain Database (http://www.ncbi.nlm.nih.gov/Structure/cdd/wrpsb.cgi), or if >30% of the gene was disrupted overall. For the genes assembled from 454 data, raw reads were mapped back against the assembled gene sequence using blastn to ensure that pseudogene calls were not the result of errors involving mononucleotide repeats or other errors due to the low-coverage nature of the data.

### Phylogenetic evaluation of horizontal transfer of the *cox1* intron

An angiosperm *cox1* intron alignment containing sequences used in previous studies^22^,^23^, including the intron from the Orobanchaceae parasite *E. virginiana*, was provided by Dr. Virginia Sanchez-Puerta. Additional Orobanchaceae *cox1* intron sequences were extracted from their best assemblies generated in this study and then manually aligned to the data set. Alignments were trimmed of poorly aligned regions with Gblocks 0.91b using relaxed parameters (b2 = half+1, b4 = 5, b5 = half). The final trimmed data set contained 958 aligned nucleotide positions and 194 intron sequences, representing 191 angiosperm species from 60 families (Table S1). The *cox1* intron alignment was then used to construct a phylogenetic tree using maximum-likelihood in PhyML 3.0^48^. A GTR+G+I model with four substitution rate categories was employed. Tree topologies, branch lengths, and rate parameters were optimized during the run. Branch support was calculated from 500 bootstrap replicates.

## Additional Information

**How to cite this article**: Fan, W. *et al*. Limited mitogenomic degradation in response to a parasitic lifestyle in Orobanchaceae. *Sci. Rep.* **6**, 36285; doi: 10.1038/srep36285 (2016).

**Publisher’s note:** Springer Nature remains neutral with regard to jurisdictional claims in published maps and institutional affiliations.

## Supplementary Material

Supplementary Information
