# Supplementary material for: Limited mitogenomic degradation in response to a parasitic lifestyle in Orobanchaceae
Source: Sci Rep. 2016 Nov 3;6:36285. doi: 10.1038/srep36285 (PMC5093741; doi:10.1038/srep36285)
Supplement: Supplementary Information [file srep36285-s1.pdf]

Limited mitogenomic degradation in response to a parasitic lifestyle in Orobanchaceae

Weishu Fan [1,2], Andan Zhu [1,2], Melisa Kozaczek [1], Neethu Shah [1,3], Natalia Pabón-Mora [4], Favio González [5], Jeffrey P. Mower [1,2]

1 Center for Plant Science Innovation, University of Nebraska, Lincoln, NE 68588, USA

2 Department of Agronomy and Horticulture, University of Nebraska, Lincoln, NE 68583, USA

3 Department of Computer Sciences and Engineering, University of Nebraska, Lincoln, NE 68588, USA

4 Instituto de Biología, Universidad de Antioquia, Apartado 1226, Medellín, Colombia

5 Facultad de Ciencias, Instituto de Ciencias Naturales, Universidad Nacional de Colombia, Apartado 7495, Sede Bogotá, Colombia

Table S1. GenBank accession numbers for all *cox1* intron sequences used in phylogenetic analysis

Family	Species	Accn. No.
Acanthaceae	Barleria prionitis	AJ247601
Acanthaceae	Justicia americana	AJ247602
Acanthaceae	Sanchezia nobilis	AJ223437
Acanthaceae	Thunbergia erecta	AJ247603
Anacardiaceae	Rhus glabra	EU281065
Apocynaceae	Alstonia plumosa	EU069541
Apocynaceae	Alyxia loesneriana	EU069542
Apocynaceae	Asclepias tuberosa	EU281054
Apocynaceae	Hoya sikkimensis	AJ247588
Apocynaceae	Neisosperma brevitubum	EU069543
Apocynaceae	Nerium oleander	AJ223421
Apocynaceae	Ochrosia elliptica	EU069544
Apocynaceae	Vinca rosea	AJ223423
Apodanthaceae	Pilostyles thurberi AZ	EU281092
Apodanthaceae	Pilostyles thurberi TX	EU281018
Aquifoliaceae	Ilex sp. Qiu 94038	AJ223429
Araceae	Alocasia cucullata	EF517193
Araceae	Alocasia gageana	EF517194
Araceae	Alocasia navicularis	EF517195
Araceae	Amorphophallus rivieri	AJ007548
Araceae	Ariopsis protanthera	EF517198
Araceae	Arisaema speciosum	EF517176
Araceae	Arisaema tortuosum	EF517177
Araceae	Arisaema triphyllum	AY009454
Araceae	Arum concinnatum	EF517179
Araceae	Arum dioscoridis	EF517180
Araceae	Arum italicum	EF517181
Araceae	Biarum davisii	EF517182
Araceae	Biarum tenuifolium	EF517183
Araceae	Caladium bicolor	EF517207
Araceae	Colocasia esculenta	EF517196
Araceae	Dracunculus canariensis	EF517184
Araceae	Dracunculus vulgaris	EF517185
Araceae	Eminium spiculatum	EF517186
Araceae	Helicodiceros muscivorus	EF517187
Araceae	Philodendron oxycardium	AJ223438
Araceae	Pinellia cordata	EF517175
Araceae	Pinellia ternata	EF517178
Araceae	Pistia stratiotes	AJ007546
Araceae	Remusatia vivipara	EF517197
Araceae	Steudnera cf. discolor	EF517200
Araceae	Steudnera discolor	EF517199
Araceae	Steudnera griffithii	EF517210
Araceae	Steudnera kerrii	EF517208
Araceae	Steudnera kerrii	EF517209
Araceae	Theriophonum infaustum	EF517202
Araceae	Typhonium albidinervum	EF517192
Araceae	Typhonium giganteum	EF517189
Araceae	Typhonium hirsutum	EF517190
Araceae	Typhonium trilobatum	EF517188
Araceae	Typhonium venosum	EF517191
Araceae	Xanthosoma mafaffa	AJ223807
Araceae	Xanthosoma sagittifolium	EF517206
Araceae	Zamioculcas zamiifolia	AJ007547
Araliaceae	Hydrocotyle rotundifolia	AJ223424
Aristolochiaceae	Aristolochia elegans	AY009431
Aristolochiaceae	Asimina triloba	AY009433
Balanophoraceae	Ombrophytum subterraneum	EU281081
Bignoniaceae	Catalpa fargesii	AJ223411
Burseraceae	Bursera simaruba	EU281030
Calceolariaceae	Calceolaria sp. IUGH	AJ247585
Celastraceae	Brexia madagascarensis	AJ223413
Clusiaceae	Montrouziera cauliflora	EU069550
Convolvulaceae	Cuscuta japonica	EU281077
Convolvulaceae	Ipomoea coccinea	EU281050
Cucurbitaceae	Citrullus lanatus	EU069546
Cucurbitaceae	Cucumis melo	EU069547
Cucurbitaceae	Cucumis metuliferus	EU069548
Cucurbitaceae	Cucumis sativus	AJ223416
Cucurbitaceae	Neoachmandra indica	EU069549
Cynomoriaceae	Cynomorium coccineum	EU281023
Cytinaceae	Cytinus ruber	EU281022
Dipterocarpaceae	Shorea talura	AJ247599
Droseraceae	Dionaea muscipula	AY600108
Ebenaceae	Diospyros virginiana	AJ223417
Ehretiaceae	Ehretia anacua	AJ247606
Ehretiaceae	Lennoa madreporoides	EU281080
Ehretiaceae	Pholisma arenarium	EU281083
Ericaceae	Pyrola secunda	AJ247582
Euphorbiaceae	Acalypha sp. Qiu95079	AJ247597
Euphorbiaceae	Codiaeum peltatum	EU069551
Euphorbiaceae	Croton alabamensis	EU281037
Euphorbiaceae	Croton sp. Qiu 94027	AJ247608
Euphorbiaceae	Euphorbia millii	AJ223418
Euphorbiaceae	Hevea brasiliensis	AJ223436
Euphorbiaceae	Hura crepitans	AJ247584
Gentianaceae	Frasera caroliniensis	EU281038
Gesneriaceae	Drymonia serrulata	AJ247579
Gesneriaceae	Nematanthus hirsutus	AJ247578
Heliotropiaceae	Heliotropium arborescens	AJ223425
Hydnoraceae	Hydnora africana	EU281079
Hydnoraceae	Prosopanche americana	EU281082
Lamiaceae	Ajuga reptans	AJ247595
Lamiaceae	Clerodendrum trichotomum	AJ223414
Lamiaceae	Lamium sp. Qiu 95015	AJ223428
Lamiaceae	Oxera sp. MVSP-2007	EU069545
Lamiaceae	Physostegia virginiana	AJ247594
Lamiaceae	Scutellaria mociniana	AJ247593
Lauraceae	Cassytha filiformis	EU281076
Lecythidaceae	Barringtonia asiatica	AJ247581
Lecythidaceae	Barringtonia racemosa	GU321958
Linaceae	Linum sp. Qiu96175	AJ247604
Loganiaceae	Strychnos spinosa	AJ247596
Loranthaceae	Dendrophthoe pentandra	EU281073
Malpighiaceae	Malpighia glabra	AJ223433
Marantaceae	Ctenanthe setosa	AY673019
Marantaceae	Maranta bicolor	AY673024
Marantaceae	Maranta leuconeura	AJ223432
Marantaceae	Monotagma laxum	AY673026
Marantaceae	Saranthe leptostachya	EU069559
Marantaceae	Saranthe sp. Kress 96-5737	AY673030
Meliaceae	Dysoxylum canalense	EU069558
Meliaceae	Melia toosendan	AJ223420
Mitrastemonaceae	Mitrastema yamamotoi	EU281021
Musaceae	Musa acuminata	AJ247609
Musaceae	Musella lasiocarpa	AY673040
Myristicaceae	Knema latericia	AJ223430
Myristicaceae	Myristica fragrans	AJ223434
Oleaceae	Jasminum floridum	EU281051
Oleaceae	Jasminum polyanthum	AJ247607

Table S1. Continued

Family	Species	Accn. No.
Opiliaceae	Lepionurus sylvestris	AJ223439
Orchidaceae	Chamorchis alpina	EF143191
Orobanchaceae	Bartsia pedicularioides	KP940490
Orobanchaceae	Castilleja paramensis	KT959112
Orobanchaceae	Epifagus virginiana	EU281078
Orobanchaceae	Lindenbergia philippensis	KT961690
Orobanchaceae	Orobanche crenata	KT961691
Orobanchaceae	Phelipanche ramosa	KT961692
Orobanchaceae	Schwalbea americana	KT961693
Paulowniaceae	Paulownia tomentosa	AJ247592
Pedaliaceae	Sesamum indicum	AJ247598
Phyllanthaceae	Breynia nivosa	AJ247605
Phyllanthaceae	Phyllanthus gneissicus	EU069552
Piperaceae	Peperomia cubensis	AF029783
Piperaceae	Peperomia griseoargentea	AF029781
Piperaceae	Peperomia obtusifolia	AF029782
Piperaceae	Peperomia polybotrya	X87336
Plantaginaceae	Aragoa abietina	EU069508
Plantaginaceae	Aragoa cundinamarcensis	EU069509
Plantaginaceae	Callitriche heterophylla	AJ247577
Plantaginaceae	Callitriche sp.	unpublished
Plantaginaceae	Digitalis purpurea	AJ223415
Plantaginaceae	Globularia punctata	EU156494
Plantaginaceae	Hebe subalpina	AJ223419
Plantaginaceae	Plantago arenaria	EU069513
Plantaginaceae	Plantago atrata	EU069536
Plantaginaceae	Sibthorpia peregrina	EU069540
Plantaginaceae	Veronica agrestis	AJ223427
Polygalaceae	Polygala sanguinea	EU281061
Polygalaceae	Polygala verticillata	unpublished
Rafflesiaceae	Rafflesia pricei	EU281020
Rafflesiaceae	Rhizanthes lowii	EU281019
Rehmanniaceae	Rehmannia glutinosa	AJ247589
Rhamnaceae	Bathiorhamnus cryptophorus	EU069557
Rhamnaceae	Frangula alnus	EU156521
Rhamnaceae	Frangula caroliniana	EU281063
Rhamnaceae	Hovenia dulcis	AJ247583
Rhamnaceae	Maesopsis eminii	EU069554
Rhamnaceae	Nesiota elliptica	EU156528
Rhamnaceae	Paliurus spina-christi	EU156525
Rhamnaceae	Phylica emirnensis	EU069555
Rhamnaceae	Rhamnella franguloides	EU156523
Rhamnaceae	Rhamnus alpina	unpublished
Rhamnaceae	Rhamnus cathartica	AJ223422
Rhamnaceae	Ziziphus ornata	EU156526
Rubiaceae	Calycosiphonia macrochlamys	EU156532
Rubiaceae	Coffea arabica	AJ247586
Rubiaceae	Ixora sp. Kew 21361	EU156535
Rubiaceae	Ixora sp. Qiu95051	AJ247587
Scrophulariaceae	Celsia arturus	AJ247590
Scrophulariaceae	Scrophularia nodosa	AJ247591

Family	Species	Accn. No.
Solanaceae	Brunfelsia jamaicensis	JF966280
Solanaceae	Hyoscyamus aureus	JF966283
Solanaceae	Hyoscyamus boveanus	JF966294
Solanaceae	Hyoscyamus desertorum	JF966293
Solanaceae	Hyoscyamus muticus	JF966292
Solanaceae	Hyoscyamus niger	JF966290
Solanaceae	Hyoscyamus pusillus	JF966291
Solanaceae	Mandragora autumnalis	JF966297
Solanaceae	Mandragora officinarum	JF966295
Solanaceae	Mandragora sp. Kew23330	JF966296
Solanaceae	Physochlaina infundibularis	JF966285
Solanaceae	Physochlaina orientalis	JF966281
Solanaceae	Przewalskia tangutica	JF966284
Symplocaceae	Symplocus paniculata	AJ223435
Urticaceae	Pilea fontana	AJ247580
Violaceae	Hybanthus sp. IND-JM3091	EU069553
Violaceae	Viola sp. Qiu95018	AJ247600
Zingiberaceae	Boesenbergia rotunda	EU069561
Zingiberaceae	Gagnepainia godefroyi	EU069564
Zingiberaceae	Globba sessiliflora	EU069565
Zingiberaceae	Hedychium coronarium	AJ223426
Zingiberaceae	Kaempferia rotunda	EU069566
Zingiberaceae	Siphonochilus decorus	AY673043

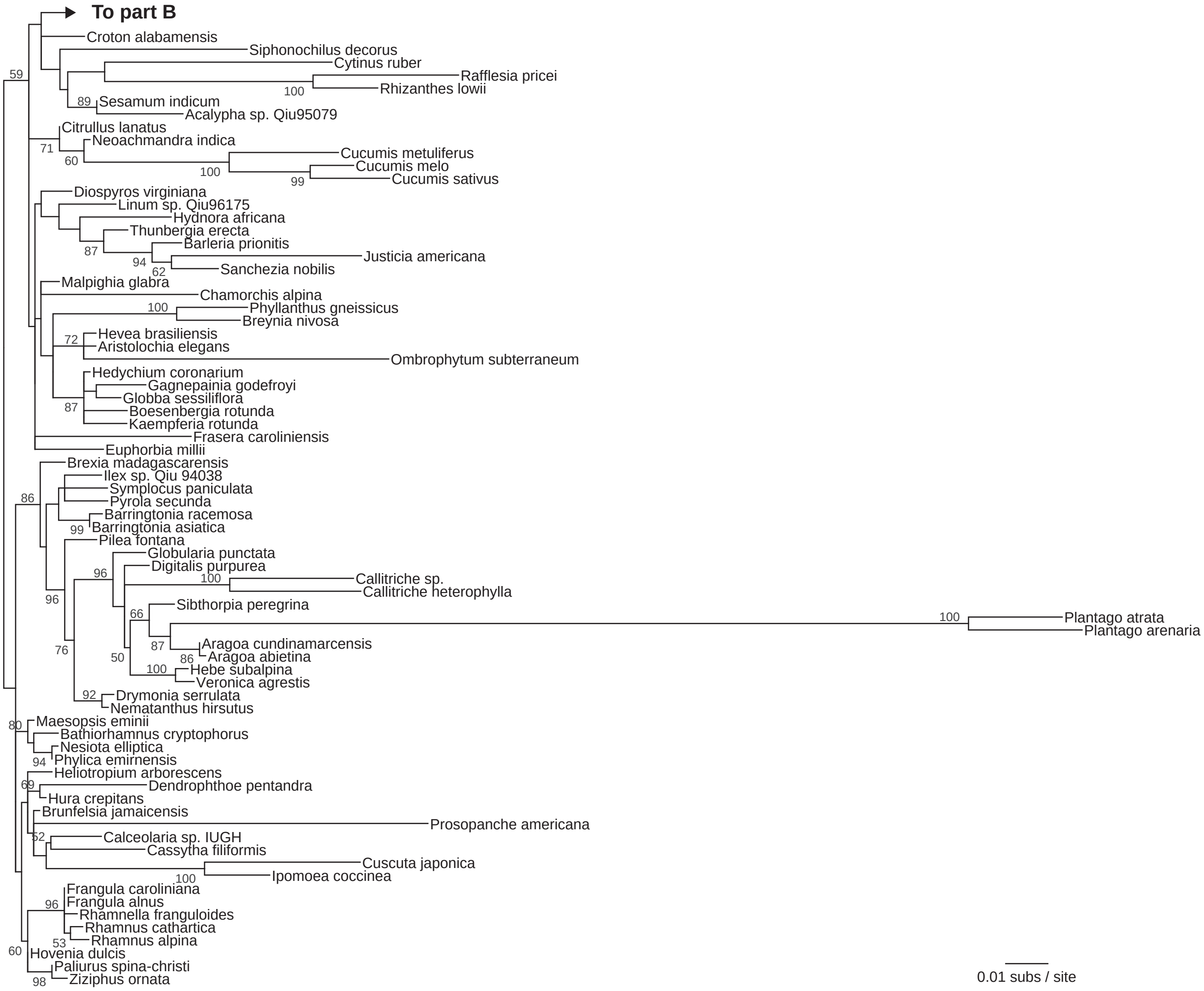

Figure S1. Labeled phylogenetic tree for the *cox1* intron. The depicted tree is identical to that in Figure 3, except that taxon names are included. The tree is split into two parts (A and B) to allow space for labeling of taxa. Orobanchaceae species are shown in bold blue text. Bootstrap values for all branches with >50% support are shown above, or in some cases below, the branch.

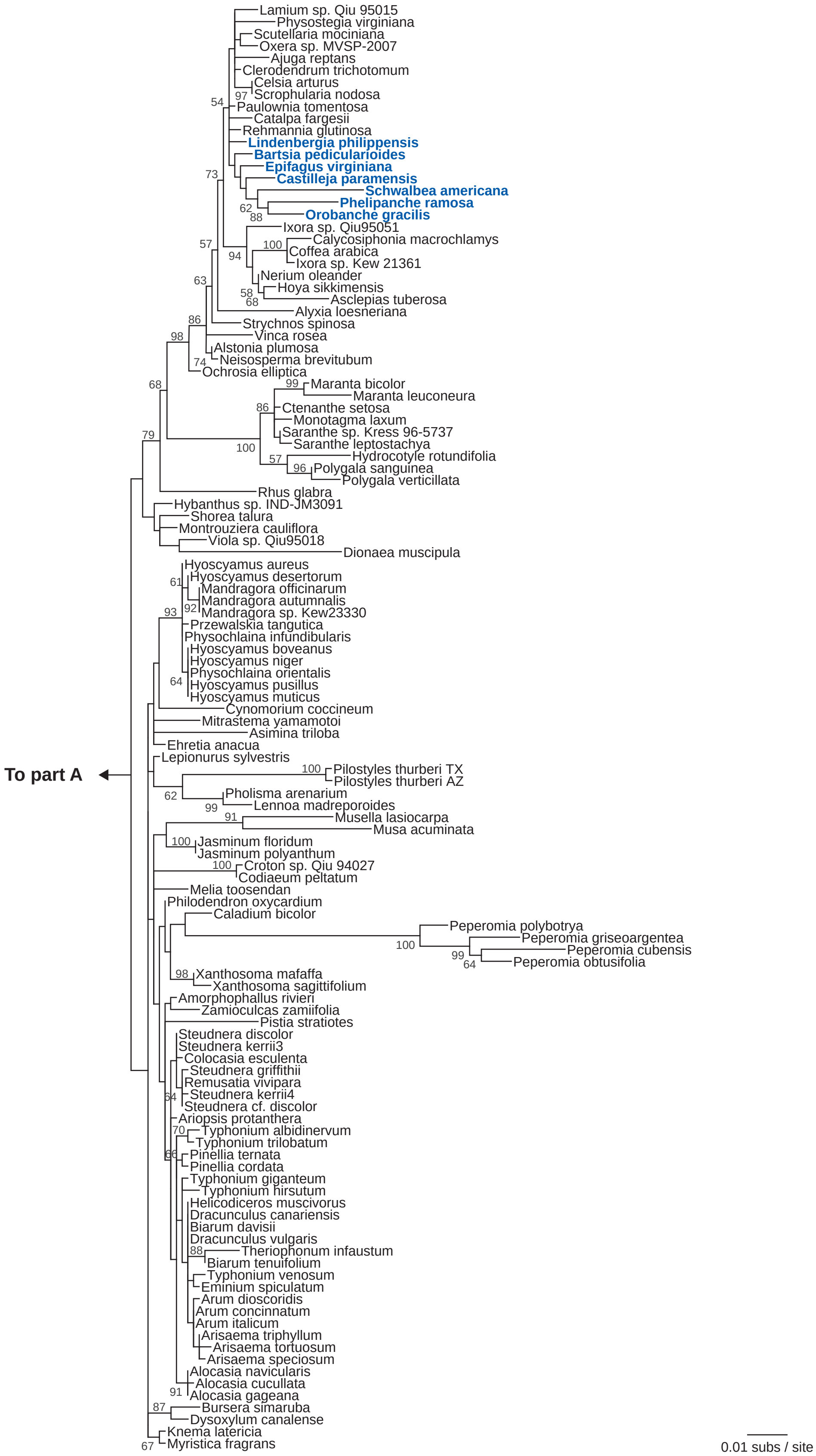

Lamium sp. Qiu 95015
Physostegia virginiana
Scutellaria mociniana
Oxera sp. MVSP-2007
Ajuga reptans
Clerodendrum trichotomum
Celsia arturus
Scrophularia nodosa
Paulownia tomentosa
Catalpa fargesii
Rehmannia glutinosa
Lindenbergia philippensis
Bartsia pedicularioides
Epifagus virginiana
Castilleja paramensis
Schwalbea americana
Phelipanche ramosa
Orobanche gracilis
Ixora sp. Qiu95051
Calycosiphonia macrochlamys
Coffea arabica
Ixora sp. Kew 21361
Nerium oleander
Hoya sikkimensis
Asclepias tuberosa
Alyxia loesneriana
Strychnos spinosa
Vinca rosea
Alstonia plumosa
Neisosperma brevitubum
Ochrosia elliptica
Maranta bicolor
Maranta leuconeura
Ctenanthe setosa
Monotagma laxum
Saranthe sp. Kress 96-5737
Saranthe leptostachya
Hydrocotyle rotundifolia
Polygala sanguinea
Polygala verticillata
Rhus glabra
Hybanthus sp. IND-JM3091
Shorea talura
Montrouziera cauliflora
Viola sp. Qiu95018
Dionaea muscipula
Hyoscyamus aureus
Hyoscyamus desertorum
Mandragora officinarum
Mandragora autumnalis
Mandragora sp. Kew23330
Przewalskia tangutica
Physochlaina infundibularis
Hyoscyamus boveanus
Hyoscyamus niger
Physochlaina orientalis
Hyoscyamus pusillus
Hyoscyamus muticus
Cynomorium coccineum
Mitrastema yamamotoi
Asimina triloba
Ehretia anacua
To part A
Lepionurus sylvestris
Pilostyles thurberi TX
Pilostyles thurberi AZ
Pholisma arenarium
Lennoa madreporoides
Musella lasiocarpa
Musa acuminata
Jasminum floridum
Jasminum polyanthum
Croton sp. Qiu 94027
Codiaeum peltatum
Melia toosendan
Philodendron oxycardium
Caladium bicolor
Peperomia polybotrya
Peperomia griseoargentea
Peperomia cubensis
Peperomia obtusifolia
Xanthosoma mafaffa
Xanthosoma sagittifolium
Amorphophallus rivieri
Zamioculcas zamiifolia
Pistia stratiotes
Steudnera discolor
Steudnera kerrii3
Colocasia esculenta
Steudnera griffithii
Remusatia vivipara
Steudnera kerrii4
Steudnera cf. discolor
Ariopsis protanthera
Typhonium albidinervum
Typhonium trilobatum
Pinellia ternata
Pinellia cordata
Typhonium giganteum
Typhonium hirsutum
Helicodiceros muscivorus
Dracunculus canariensis
Biarum davisii
Dracunculus vulgaris
Theriophonum infaustum
Biarum tenuifolium
Typhonium venosum
Eminium spiculatum
Arum dioscoridis
Arum concinnatum
Arum italicum
Arisaema triphyllum
Arisaema tortuosum
Arisaema speciosum
Alocasia navicularis
Alocasia cucullata
Alocasia gageana
Bursera simaruba
Dysoxylum canalense
Knema latericia
Myristica fragrans
0.01 subs / site

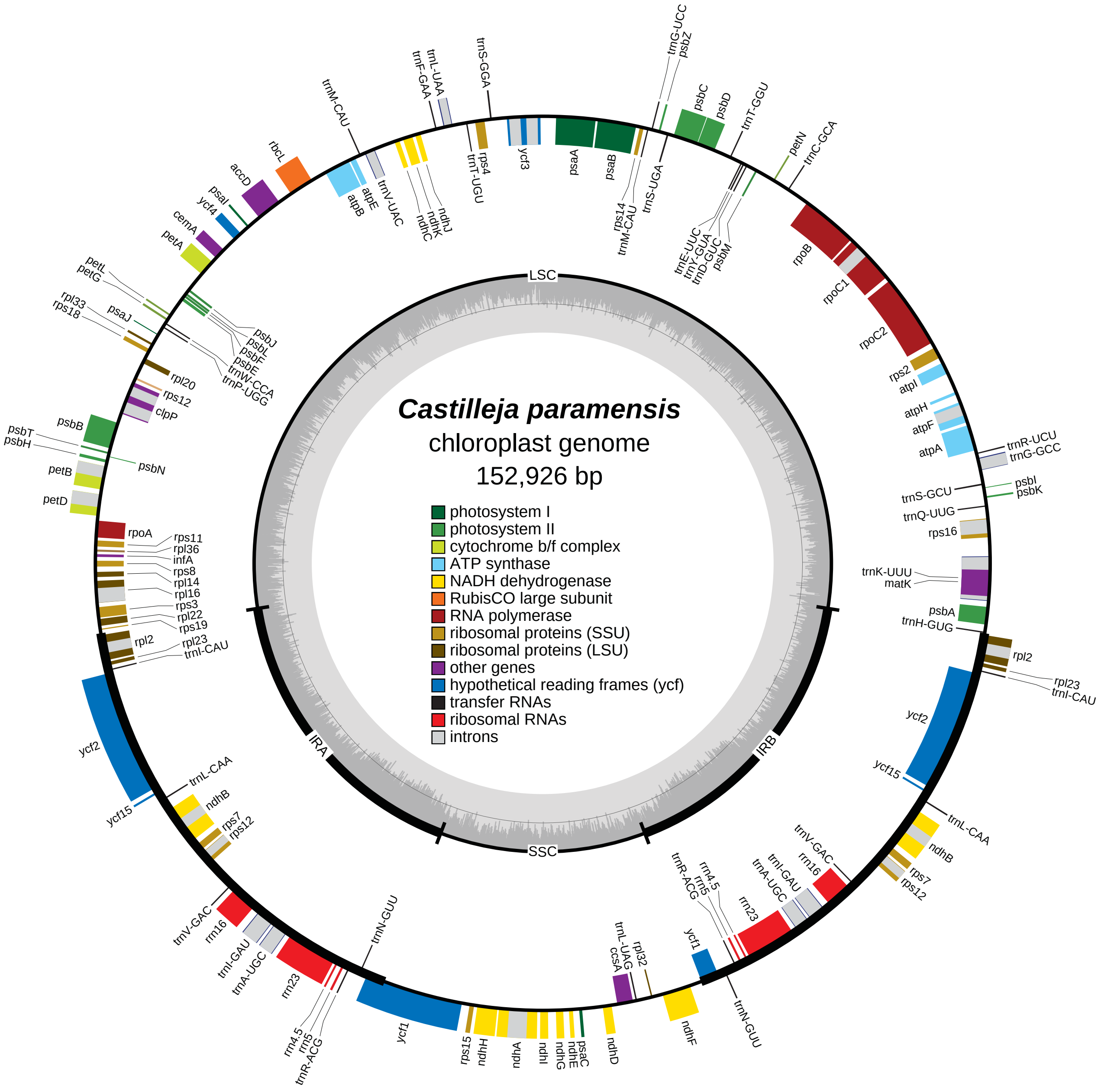

Figure S2. Circular genome map of the *Castilleja paramensis* plastome. Outer genes are transcribed counter-clockwise; inner genes are transcribed clockwise. Gene and intron colors correspond to the functional categories listed in the key. On the inner circle is shown GC content and the location of the inverted repeats (IRA, IRB) and single-copy regions (SSC, LSC). The map was drawn with OgDraw (http://ogdraw.mpimp-golm.mpg.de/).

	Orobanchaceae					Related Asterids		
	Cpa	Sam	Cde	Cam	Lph	Bhy	Nta	Dca
**Genes**								
accD	●	●	●	●	●	●	●	●
atpA	●	●	○	○	●	●	●	●
atpB	●	●	○	○	●	●	●	●
atpE	●	●	○	○	●	●	●	●
atpF	●	●	○	○	●	●	●	●
atpH	●	●	○	○	●	●	●	●
atpI	●	●	○	○	●	●	●	●
ccsA	●	●	○	○	●	●	●	●
cemA	●	●	○	○	●	●	●	●
clpP	●	●	●	●	●	●	●	●
infA	●	●	●	Ψ	●	●	Ψ	●
matK	●	●	●	●	●	●	●	●
ndhA	●	Ψ	○	○	●	●	●	●
ndhB	●	●	Ψ	Ψ	●	●	●	●
ndhC	●	●	○	○	●	●	●	●
ndhD	Ψ	Ψ	○	○	●	●	●	●
ndhE	●	●	○	○	●	●	●	●
ndhF	Ψ	Ψ	○	○	●	●	●	●
ndhG	●	Ψ	○	○	●	●	●	●
ndhH	Ψ	●	○	○	●	●	●	●
ndhI	●	○	○	○	●	●	●	●
ndhJ	Ψ	Ψ	○	○	●	●	●	●
ndhK	●	Ψ	○	○	●	●	●	●
petA	●	●	○	○	●	●	●	●
petB	●	●	○	○	●	●	●	●
petD	●	●	○	○	●	●	●	●
petG	●	●	○	○	●	●	●	●
petL	●	●	○	○	●	●	●	●
petN	●	●	○	○	●	●	●	●
psaA	●	●	○	○	●	●	●	●
psaB	●	●	○	○	●	●	●	●
psaC	●	●	○	○	●	●	●	●
psaI	●	●	○	○	●	●	●	●
psaJ	●	●	○	○	●	●	●	●
psbA	●	●	Ψ	Ψ	●	●	●	●
psbB	●	●	○	○	●	●	●	●
psbC	●	●	Ψ	○	●	●	●	●
psbD	●	●	Ψ	○	●	●	●	●
psbE	●	●	Ψ	○	●	●	●	●
psbF	●	●	Ψ	○	●	●	●	●
psbH	●	●	○	○	●	●	●	●
psbI	●	●	Ψ	○	●	●	●	●
psbJ	●	●	Ψ	○	●	●	●	●
psbK	●	●	Ψ	○	●	●	●	●
psbL	●	●	○	○	●	●	●	●
psbM	●	●	●	○	●	●	●	●
psbN	●	●	○	○	●	●	●	●
psbT	●	●	○	○	●	●	●	●
psbZ	●	●	○	○	●	●	●	●
rbcL	●	●	Ψ	○	●	●	●	●
rpl2	●	●	●	●	●	●	●	●
rpl14	●	●	●	●	●	●	●	●
**Genes (continued)**								
rpl16	●	●	●	●	●	●	●	●
rpl20	●	●	●	●	●	●	●	●
rpl22	●	●	●	Ψ	●	●	●	●
rpl23	●	●	Ψ	Ψ	●	●	●	●
rpl32	●	●	●	○	●	●	●	●
rpl33	●	●	●	●	●	●	●	●
rpl36	●	●	●	●	●	●	●	●
rpoA	●	●	○	○	●	●	●	●
rpoB	●	●	○	○	●	●	●	●
rpoC1	●	●	○	○	●	●	●	●
rpoC2	●	●	Ψ	○	●	●	●	●
rps2	●	●	●	●	●	●	●	●
rps3	●	●	●	●	●	●	●	●
rps4	●	●	●	●	●	●	●	●
rps7	●	●	●	●	●	●	●	●
rps8	●	●	●	●	●	●	●	●
rps11	●	●	●	●	●	●	●	●
rps12	●	●	●	●	●	●	●	●
rps14	●	●	●	●	●	●	●	●
rps15	●	●	●	○	●	●	●	●
rps16	●	●	●	Ψ	●	●	●	●
rps18	●	●	●	●	●	●	●	●
rps19	●	●	●	●	●	●	●	●
ycf1	●	●	Ψ	●	●	●	●	●
ycf2	●	●	●	●	●	●	●	●
ycf3	●	●	○	○	●	●	●	●
ycf4	●	●	Ψ	○	●	●	●	●
**Introns**								
atpF-i1	●	●	x	x	●	●	●	●
clpP-i1	●	●	●	●	●	●	●	●
clpP-i2	●	○	●	●	●	●	●	●
ndhA-i1	●	x	x	x	●	●	●	●
ndhB-i1	●	●	x	x	●	●	●	●
petB-i1	●	●	x	x	●	●	●	●
petD-i1	●	●	x	x	●	●	●	●
rpl2-i1	●	●	●	●	●	●	●	●
rpl16-i1	●	●	●	●	●	●	●	●
rps12-i1	●	●	●	●	●	●	●	●
rps12-i2	●	●	●	●	●	●	●	●
rps16-i1	●	●	●	x	●	●	●	●
rpoC1-i1	●	●	x	x	●	●	●	●
trnA-i1	●	●	●	●	●	●	●	●
trnG-i1	●	●	●	x	●	●	●	●
trnI-i1	●	●	●	x	●	●	●	●
trnK-i1	●	●	●	●	●	●	●	●
trnL-i1	●	●	x	●	●	●	●	●
trnV-i1	●	●	●	x	●	●	●	●
ycf3-i1	●	●	x	x	●	●	●	●
ycf3-i2	●	●	x	x	●	●	●	●
Total genes	75	72	26	21	79	79	78	79
Total introns	21	19	12	9	21	21	21	21

Figure S3. Plastome gene and intron content in plastomes from Orobanchaceae and selected asterids. Genes and introns present in each genome are marked with a filled circle ("●"). Lost genes and introns ("○"), pseudogenes ("ψ"), and missing introns due to loss or pseudogenization of the host gene ("x") are shaded gray. Cpa = *Castilleja paramensis*; Sam = *Schwalbea americana*; Cde = *Cistanche deserticola*; Cam = *Conophilis americana*; Lph = *Lindenbergia philippensis*; Bhy = *Boea hygrometrica*; Nta = *Nicotiana tabacum*; Dca = *Daucus carota*.
